# Biomedical relation extraction with knowledge base–refined weak supervision

**DOI:** 10.1093/database/baad054

**Published:** 2023-07-26

**Authors:** Wonjin Yoon, Sean Yi, Richard Jackson, Hyunjae Kim, Sunkyu Kim, Jaewoo Kang

**Affiliations:** Department of Computer Science and Engineering, Korea University, 145 Anam-ro, Seongbuk-gu, Seoul 02841, South Korea; Department of Computer Science and Engineering, Korea University, 145 Anam-ro, Seongbuk-gu, Seoul 02841, South Korea; AstraZeneca UK, 1 Francis Crick Ave, Trumpington, Cambridge CB2 0AA, UK; Department of Computer Science and Engineering, Korea University, 145 Anam-ro, Seongbuk-gu, Seoul 02841, South Korea; Department of Computer Science and Engineering, Korea University, 145 Anam-ro, Seongbuk-gu, Seoul 02841, South Korea; AIGEN Sciences Inc., 25 Ttukseom-ro 1-gil, Seongdong-gu, Seoul 04778, South Korea; Department of Computer Science and Engineering, Korea University, 145 Anam-ro, Seongbuk-gu, Seoul 02841, South Korea; AIGEN Sciences Inc., 25 Ttukseom-ro 1-gil, Seongdong-gu, Seoul 04778, South Korea

## Abstract

Biomedical relation extraction (BioRE) is the task of automatically extracting and classifying relations between two biomedical entities in biomedical literature. Recent advances in BioRE research have largely been powered by supervised learning and large language models (LLMs). However, training of LLMs for BioRE with supervised learning requires human-annotated data, and the annotation process often accompanies challenging and expensive work. As a result, the quantity and coverage of annotated data are limiting factors for BioRE systems. In this paper, we present our system for the BioCreative VII challenge—DrugProt track, a BioRE system that leverages a language model structure and weak supervision. Our system is trained on weakly labelled data and then fine-tuned using human-labelled data. To create the weakly labelled dataset, we combined two approaches. First, we trained a model on the original dataset to predict labels on external literature, which will become a model-labelled dataset. Then, we refined the model-labelled dataset using an external knowledge base. Based on our experiment, our approach using refined weak supervision showed significant performance gain over the model trained using standard human-labelled datasets. Our final model showed outstanding performance at the BioCreative VII challenge, achieving 3rd place (this paper focuses on our participating system in the BioCreative VII challenge).

**Database URL:** http://wonjin.info/biore-yoon-et-al-2022

## Introduction

Biomedical relation extraction (BioRE) is a text mining task to automatically extract relations between entities in biomedical literature. The BioRE task can be used as core components for building biomedical knowledge graphs or drug response in cancer (1–3).

Recently, supervised learning and large-scale biomedical language models have contributed substantially to advancements in BioRE research (4–7). Models trained using these approaches can be considerably influenced by the quality and the quantity of training data. However, creating datasets for biomedical natural language processing (NLP) tasks is a challenging task owing to the complexity of biomedical information in the literature, which often results in low inter-annotator agreement (8). Consequently, the annotation costs for BioRE datasets can be higher than those for general domain datasets, making it difficult to build a high-quality large-scale BioRE dataset. Accordingly, researchers have attempted to explore approaches for data augmentation. Such an approach involves leveraging external resources by creating distantly supervised datasets with domain-specific knowledge bases (KBs) (9–11) and utilizing model-generated labels to augment the existing dataset (12, 13).

One of the data augmentation approaches, i.e. distantly labelling biomedical relations using KBs (14, 15), enables the automatic construction of large-scale datasets. However, this method can produce noisy labels, as most KBs are not specifically designed for NLP applications. Since KBs typically lack example sentences and entity location annotations within sentences, distant supervision relies on the assumption that if two entities with a known relation in a KB appear in a sentence, the sentence is labelled as having that relation. (Furthermore, a significant portion of biomedical relations are complex. These relations are not limited to two entities, nor can they be described in a single sentence within biomedical literature. Recent datasets, such as BioRED (16), tackle more complex settings for BioRE (please refer the Related works section). Nonetheless, this paper focuses on the more straightforward settings of DrugProt, in which we extract relations between drugs and proteins within a single sentence.) This assumption can lead to erroneous labels: a sentence containing a relation may be labelled as a negative example if the relation is not listed in the KB (False Negative), and a sentence may be labelled as a positive example even if the two entities merely coexist within it without semantically indicating a relation (False Positive). For example, the relation ‘acetaldehyde increases alpha 2(I) collagen’ is listed in the KB [we used the Comparative Toxicogenomics Database (CTD)], but this relation is not directly expressed in the sentence ‘Effects of acetaldehyde on nuclear protein binding to the nuclear factor I consensus sequence in the alpha 2(I) collagen promoter’. Simple distant labelling without any additional approach will mark the entities in the example sentence as having a relationship.

Another approach, known as (model-label-based) weak supervision, involves using pseudo-labelled datasets, or ‘weak labels’, which are generated from predictions made by a model trained on a small, high-quality dataset. (In the rest of this paper, we will denote model-label-based weak supervision as simply ‘weak supervision’.) The weak supervision approach has the potential to generate large-scale datasets with more diverse sentence and extended relation patterns than the original dataset (17). This method has been shown to be effective in other research tasks in biomedical NLP (12, 13). However, labelling bias noises in the original dataset inherit the weakly labelled datasets, and the limited coverage of knowledge in the original dataset adds noises to the augmented datasets (12).

We propose a system for BioRE that combines some of the advantages of both weakly supervised learning and the utilization of external resources. Our approach consists of three phases: building a large-scale augmented dataset, pretraining a transformer model using the augmented dataset and finally fine-tuning the model with the original human-annotated dataset (such as DrugProt (18) or ChemProt (19)). The first phase is the step of preparing a training dataset for our system. Generation of weakly labelling corpus and filtering of the generated dataset using KB are performed in this phase. Specifically, we train a model using the DrugProt dataset and predict relations in the selected MEDLINE articles. Predicted relations are then compared with drug–protein relation triples in the KB and are dropped if the labels from two sources disagree. The second and third phases are about how to effectively train our system using weakly labelled and human-labelled datasets. We trained a transformer-based sequence classification model on the augmented dataset and transferred the trained model weights to fine-tune the model using the original dataset, DrugProt.

The contributions of this paper are 3-fold. First, we propose a system for the BioRE using a weakly supervised data augmentation approach, which showed near state-of-the-art performance at the DrugProt challenge. Second, we perform an in-depth analysis of the predictions of our model and showed the robustness of our model on relatively scarce type relations. Finally, we have made our resources publicly available, including trained model weights and automatic annotations on the 31 129 681 drug–protein entity pairs, of which 6 355 642 pairs are predicted as potential relations.

## Materials and Methods

**Figure 1.**
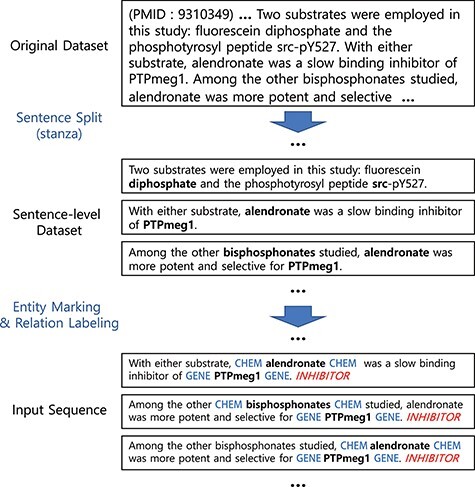
Preprocessing steps. Entity markers wrap entities in input sequences.

**Figure 2.**
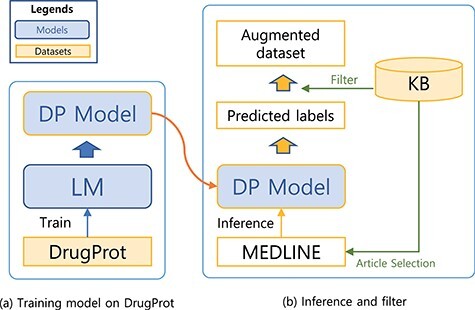
A system for building a large-scale augmented dataset: (**a**) training model on DrugProt and (**b**) inference and filter.

**Figure 3.**
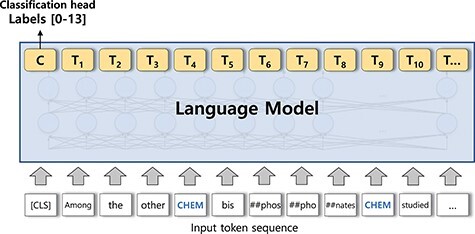
A sequence classification model. The output of the [CLS] token is used for the classification head.

In this section, we describe elements in our system for BioRE. We first describe our preprocessing steps (Figure 1), for preparing input sequences in our experiments. Then, we describe methods to build a large-scale augmented dataset (Figure 2), the first phase of our system. Finally, we describe our sequence classification model, the main part of the system, which classifies the type of the relation for an entity pair in the given sentence (Figure 3).

### Preprocessing

We used an identical structure for the neural network model throughout all three phases of our system. (We will describe our neural network model, or sequence classification model, in the later part of the Methods section.) This allowed us to maintain a unified input sequence format across all three phases.

Figure 1 illustrates our preprocessing steps and examples. Our preprocessing starts by splitting documents (in our case, abstracts) into sentences using the Stanza library (20, 21). We then mark the entities by inserting special tokens to wrap before and after the drug/chemical and gene/protein entities. Note that whether to use a different token for each type or whether to wrap or replace the entity can be a hyper-parameter to choose.

### Building a large-scale augmented dataset

Training a model that can generalize to diverse relation expression patterns is a key factor to get a robust model. In order to expose our model to diverse patterns, we augmented the dataset using the predictions of the model as weakly labelled data. In order to filter noises, we refined the weak labels with KB.

The steps for preparing the source (unlabelled) dataset for the augmented dataset is analogous to the preprocessing steps for the DrugProt dataset (Figure 1). First, we collect documents that appear as reference articles in a KB for a targeting relation type. Then, we split the abstracts into sentences using the Stanza library. In the third step, we recognize Gene- and Chemical-type entities [also known as named entity recognition (NER)] within the sentences. To achieve this, we used both neural-network-based and dictionary-based methods for the NER. Specifically, we used BERN (22), a BioBERT-based online NER tool. Then, we have also utilized the dictionaries of the KB and external source HUGO Gene Nomenclature Committee gene set (23)) for additional steps to detect entities. Finally, we attached entity markers at the beginning and end of the entity.

We first trained a sequence classification model with the DrugProt dataset and used the trained model to predict the potential label for the unlabelled dataset. A KB was used to check the validity of predicted labels. Since the labelling schema for the dataset and KB is different, we used KB to only check the presence of the relation between the two entities. For example, if a prediction for a sequence indicates that there is a relation between the entity pair in the sequence, the predicted label will only be used for the augmented dataset if the entity pair is listed as having a relation in the KB. Sequences without agreement are dropped. Although this filtering may drop potentially useful samples, we believe that the drawbacks are marginal. With a large weakly labelled dataset, we can still obtain sufficient examples to capture diverse relation patterns. Moreover, by reducing noise through the filtering process, we expect to improve the precision of our model. Therefore, we consider the trade-off to be beneficial in improving the overall model performance.

### Sequence classification model

Our sequence classification model applied a straightforward method. Following BERT (24) and BioBERT (4), we used the output of special token, [CLS], as the sentence representation for the classification head. A linear classification layer was used. Different from the experiments of BioBERT, we did not anonymize entities and used different entity markers for entity types and for the start and end of the entity. We registered four entity markers to the vocabulary file. We discovered through ablation experiments that using independent entity markers, registering markers and non-anonymized entities showed the best performance, showing ~0.5–1% performance gain for each element.

## Experimental settings

In this section, we describe the implementation details of our study. We provide an overview of the datasets used, including statistics and details on their preparation, as well as our experimental settings, which include the parameters and techniques used to train and evaluate our models. Furthermore, we analyse the computational costs of our experiments.

### Biomedical resources and datasets

#### Knowledge base

For our KB source, we used the chemical–gene interactions data from the CTD (25). CTD is a comprehensive database of scientific entities such as drugs/chemicals, proteins and diseases, along with manually curated relations between them and the identifiers (specifically, PMIDs) of supporting documents. Please note that although CTD contains PMIDs of supporting documents, it does not provide enough detail to build a distantly labelled dataset, causing noises.

We collected documents from the CTD—Chem/Gene database that were cited as supporting evidence for relationships between drugs/chemicals and genes/proteins (we used August 2021 version). To maintain the integrity of the evaluation, we excluded documents that were also present in the development dataset of DrugProt. After filtering, we collected 68 392 documents, of which ~1% existed in both the development set and the KB and were excluded from our augmented dataset resources. These documents were split into 686 885 sentences. After the basic data-cleaning process, the total number of chemical and protein entity combinations was ~1.26 million (352 000 combinations were excluded). Of these, 875 000 combinations agreed with our model predictions and were used to create the augmented dataset, while 387 000 combinations were filtered out owing to non-agreement with the model predictions.

#### DrugProt dataset and augmented dataset

The DrugProt dataset is a human-annotated dataset on relations between chemical/drug and gene/protein entities (18). Similar to the previous challenge dataset CHEMPROT of BioCreative VI (19), the DrugProt dataset provides valuable datasets for training and evaluating automatic prediction models of relation extraction.

Table 1 summarizes the statistics for the DrugProt dataset (18) and the augmented dataset. The DrugProt dataset itself is a rich source for training BioRE models and has 64 779 training data points. When we apply our system, we were able to build an augmented dataset of 875 350 training data points, which is ~13.5 times larger than the original DrugProt dataset. Table 3 shows the statistics of the relations in the DrugProt dataset. Since the relation classes are skewed, some classes have a small number of training data points.

**Table 1.** Statistics of the datasets: the number of data points for the original dataset and augmented dataset

	Number of input sequences	
Dataset	Train	Development
Original (DrugProt)	64 779	13 480
Augmented dataset	875 350	

#### Hyper-parameters and language models

Our hyper-parameter settings and pretrained weights of language models, or backbone models, are selected using the performance of the model on the development dataset. For accurate comparison between the settings and to minimize the effect of randomness by the parameter initialization step, we conducted multiple independent runs (mostly 10 independent runs unless otherwise stated) using different random seeds and used the average performance for the selection.

**Table 2.** Comparisons on the language models pretrained on biomedical literature

Language model	Architecture	No. of parameters	Corpora for training (self-supervision)	Vocab source	Vocab size	Case
BioBERT	BERT	108 million (base)	PubMed (continued from BERT)	Wiki+books	28 996	Cased
PubMedBERT	BERT	108 million (base)	PubMed	PubMed	30 522	Uncased
BioLM$_\textrm{LARGE}$	RoBERTa	355 million (large)	PubMed, PMC and MIMIC-III	PubMed	50 008	Cased

*Notes:* BioBERT, PubMedBERT and BioLM in the ‘Language model’ column denotes checkpoints named ‘biobert-base-cased-v1.1’, ‘BiomedNLP-PubMedBERT-base-uncased-abstract’ and ‘RoBERTa-large-p.m.-M3-Voc-hf’ from the official resource links of the original authors.

Uncased in the ‘Case’ column means that all the characters in the input string are lower-cased in the preprocessing steps, whereas Cased means that the original string is preserved.

Our hyper-parameter searching was mainly focused on four variables: learning rate, training epochs, mini-batch size and special tokens for marking entities. A learning rate was selected among 5e-5, 2e-5, 1e-5 and 5e-6. We used a linear learning rate scheduler with a warm rate of 0.05 which means that the scheduler dacays the learning rate after the warm-up steps and linearly decreases it to 0 when the training reaches the max iteration steps. For training epochs, we monitored the training steps using the average *F*1-score (averaged across five independent experiments for the development experiments and 10 for the test, or challenge, experiments). Mini-batch sizes of 16 and 32 were used for training the models on the DrugProt dataset. We explored four different methods of marking entities as follows:

- No marking (original string): ‘Alendronate’ was a slow-binding inhibitor of ‘PTPmeg1’.
- Masking entities (replace with marker): ‘CHEM’ was a slow-binding inhibitor of ‘GENE’.
- Marking with entity types: ‘CHEM Alendronate CHEM’ was a slow-binding inhibitor of ‘GENE PTPmeg1 GENE’.
- Marking with entity types with different markers in-front and after the entity: ‘CHEM-S Alendronate CHEM-E’ was a slow-binding inhibitor of ‘GENE-S PTPmeg1 GENE-E’.

Please note that the markers are registered as special tokens on the language model vocab lists. Hence, the embeddings of the marker tokens are trainable parameters. Based on our experiments, Method (iv) showed the best performance. For the experiments throughout this study, we marked entities using Method (iv).

Language model candidates were BioBERT$_\textrm{BASE}$ (4), PubMedBERT (5) and BioLM$_\textrm{LARGE}$ (RoBERTa-large-PM-M3-Voc) (7). All three models are pretrained in the biomedical domain but show variations in details (Table 2). BioBERT and PubMedBERT are based on BERT and BASE size models, whereas BioLM$_\textrm{LARGE}$ is based on the RoBERTa structure and LARGE size model where the number of parameters is three times larger than the former models.

#### Utilizing training data and development data as training material

For our challenge participation, we trained some of our models (marked as Train+Dev in Table 5) using the development dataset. First, we merged the training dataset and development dataset. Next, we split the merged dataset into 10 partitions. Then, we created 10 reorganized train–develop dataset pairs by pairing a development set from one partition with the new training set derived from the other nine partitions. We trained 10 models with the pairs. When combined with the ensemble method, we expect that this approach can virtually expand the size of training data, which is proven to be beneficial for the performance in the later part of this paper (see the Results section).

### Computations costs

#### Generating the weakly supervised dataset

Preprocessing steps and the prediction steps took less than 12 hours using a machine with one central processing unit (CPU) (16 cores) and one graphics processing unit (GPU) (TITAN RTX 24GB).

#### Inference

Based on our experiments on the large-scale text mining subtrack (large track) dataset of ~2.3 million articles (~33 million sentences), preprocessing took ~3 h and 15 min with 15 parallel processes (single-thread process) and the inference took 6 h and 30 min with a batch size of 512 (1 GPU with 24 GB GPU memory).

In total, the computation required ~10 h * 15 machines (1 CPU, 1 GPU) = 150 unit-node h. This means that our model can predict relations in one article in 0.23 s (4.2 articles per second).

**Table 3.** Statistics of the relation types in the DrugProt dataset: the number of data points and its proportion for each class

Type	Train	Development
INHIBITOR	5392 (31.3%)	1152 (30.6%)
DIRECT-REGULATOR	2250 (13.0%)	458 (12.2%)
SUBSTRATE	2003 (11.6%)	495 (13.2%)
ACTIVATOR	1429 (8.3%)	246 (6.5%)
INDIRECT-UPREGULATOR	1379 (8.0%)	302 (8.0%)
INDIRECT-DOWNREGULATOR	1330 (7.7%)	332 (8.8%)
ANTAGONIST	972 (5.6%)	218 (5.8%)
PRODUCT-OF	921 (5.3%)	158 (4.2%)
PART-OF	886 (5.1%)	258 (6.9%)
AGONIST	659 (3.8%)	131 (3.5%)
AGONIST-ACTIVATOR	29 (0.2%)	10 (0.3%)
SUBSTRATE_PRODUCT-OF	25 (0.1%)	3 (0.1%)
AGONIST-INHIBITOR	13 (0.1%)	2 (0.1%)

## Results and discussion

In this section, we discuss our experimental results for the development dataset and the test data submission for the challenge. For the test dataset, we report scores received from the challenge organizers.

### Performance of the model on the development dataset

**Figure 4.**
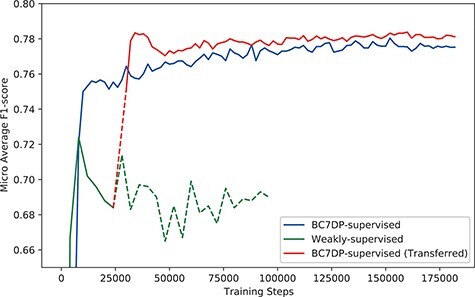
The performance (*F*1-score) of models by the total training steps. The solid line (BC7DP-supervised) represents the performance of a model trained using the original dataset. The line starting with dotted line and soon changed into solid line, denoted as BC7DP-supervised (Transferred), represents the performance of our system. The line strarting with solid line but soon changed into dotted line, denoted as Weakly-supervised, represents a model trained only on weakly supervised datasets (without the third phase of our system). Note that the solid part of BC7DP-supervised (Transferred) linestarts at the 28 000th step of the Weakly-supervised line as it is transferred from the 28 000th step of the weakly supervised only model. For the BC7DP-supervised and BC7DP-supervised (Transferred), *F*1-scores are averaged across five independent runs.

Figure 4 shows the micro-averaged *F*1-score of the plain sequence classification model trained on the BC7DP dataset, the model trained solely on our KB-refined weakly labelled dataset and our full system (denoted as BC7DP-supervised (Transferred)). Note that the performance of our model (full system) is starting from 28 000 step point since the model is initiated from the 28 000th step of the weakly supervised model. To enhance the statistical robustness of our report, we plotted the micro-averaged *F*1 scores from five independent runs, each with different random seeds. Table 4 presents the best performance of our system on the DrugProt development dataset. We saved the checkpoints with 2000, 4000 or 10 000 intervals and evaluated the saved checkpoints to find the best models. Experiments in this subsection report performance on BioLM$_\textrm{LARGE}$ as the backbone model.

**Figure 5.**
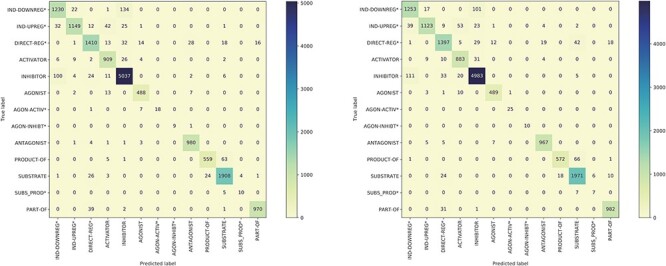
The confusion matrix of the model trained using the BC7DP dataset (left) and our proposed method (right) on the development dataset.

Figure 5 presents the confusion matrices for the model trained on the BC7DP dataset and our system. The figure highlights the skewed nature of the model predictions, illustrating the challenging aspects of the relation prediction task. Please note that predictions labelled as ‘None’ are omitted from the matrices to enhance visibility. Both matrices represent the best results achieved by each model (corresponding to the same checkpoints as in Table 4).

For our challenge participation, we explored the potential performance gain of the ensemble method. In our experiment with the ensemble method where 10 models are ensembled, we observed an increase of 1.5% (*F*1), showing 0.789 and 0.795 for the ‘1-RoBERTaLarge’ (runID 1) model and the ‘3-RoBERTaLarge_CTD’ (runID 3) model, respectively, on develop set.

**Table 4.** The performance of the model on the development dataset

	Baseline (152 000)			Our (160 000)			Difference		
	Prec (%)	Recall (%)	*F*1 (%)	Prec (%)	Recall (%)	*F*1 (%)	Prec (%)	Recall (%)	*F*1 (%)
INDIRECT-DOWNREGULATOR	75.56	75.72	75.64	77.52	75.30	76.36	1.96	-0.42	0.72
INDIRECT-UPREGULATOR	80.84	74.26	77.30	79.98	73.78	76.76	−0.86	−0.48	−0.54
DIRECT-REGULATOR	70.32	62.24	65.84	71.12	61.36	65.86	0.80	-0.88	0.02
ACTIVATOR	76.24	72.76	74.32	77.28	71.62	74.34	1.04	−1.14	0.02
INHIBITOR	84.58	86.64	85.58	86.80	86.04	86.42	2.22	−0.60	0.84
AGONIST	82.12	72.22	76.80	80.48	72.82	76.44	−1.64	0.60	−0.36
AGONIST-ACTIVATOR	96.66	48.00	63.94	100.00	48.00	64.78	3.34	0.00	0.84
AGONIST-INHIBITOR	66.70	100.00	80.00	66.70	100.00	80.00	0.00	0.00	0.00
ANTAGONIST	93.02	90.36	91.64	92.26	91.92	92.10	−0.76	1.56	0.46
PRODUCT-OF	63.42	69.76	66.26	68.24	70.40	69.24	4.82	0.64	2.98
SUBSTRATE	73.86	77.30	75.52	71.02	76.58	73.70	−2.84	−0.72	−1.82
PRODUCT-OF	66.70	66.70	66.70	60.02	60.02	59.44	−6.68	−6.68	−7.26
PART-OF	71.60	77.60	74.38	73.20	75.66	74.34	1.60	−1.94	−0.04
Micro-average	78.22	77.86	78.04	79.18	77.30	78.22	0.96	−0.56	0.18%

*Notes:* We report the statistics of five independent runs using different random seeds. INHIBITOR, DIRECT-REGULATOR and SUBSTRATE are the most abundant types of relation. This is the best performance across the training steps. Please also refer to Table 3 for detailed statistics.

### Results on the test data submission (BioCreative VII challenge)

**Table 5.** The performance of the model on test data (DrugProt challenge results)

	Settings			*F1 Performance (test)*				Recall Performance (Test)			
runID	Augumented	Data	#Models	*F*1 % (All)	*F*1 % (INHI)	*F*1 % (DIR)	*F*1 % (SUBS)	Rec % (All)	Rec % (INHI)	Rec % (DIR)	Rec % (SUBS)
1	X	Train	10	78.53	87.45	69.75	67.94	78.13	86.86	66.66	63.48
2	X	Train+Dev	10	78.93	87.78	66.74	68.27	78.16	88.20	62.47	64.20
3	O	Train	5	78.38	87.44	67.85	68.78	79.08	87.82	66.66	67.06
4	O	Train+Dev	5	78.61	87.51	69.07	69.25	77.93	88.01	64.56	66.10
5	O	Train	10	78.36	86.96	67.72	70.25	78.08	86.96	64.56	67.06

*Notes:* Evaluated by the organizers (leaderboard). The backbone model (language model) for all submissions was BioLM$_\textrm{LARGE}$. Augumented in the column denotes that the model is pretrained on the augmented dataset. #Models denotes the number of models used for the ensemble method. The Data column denotes the training materials (e.g. Train + Dev means that the model is trained on the merged dataset of the training dataset and development dataset). INHI, DIR and SUBS denotes the scores for INHIBITOR, DIRECT-REGULATOR and SUBSTRATE, which are the most abundant types of relation.

Table 5 shows the performance of our models for the main track of the challenge. For evaluating our model on test data (main track submission), we used ensembles of models. For runID 1 and 2, we trained 10 models without the augmented dataset. For runID 3 and 4, we trained five models, which were also pretrained on the augmented dataset.

We used five models for runIDs 3 and 4, the submissions using augmented data, due to the lack of time to train the models. This may have led to a relatively lower performance of our augmented dataset models. The fact that augmented dataset models perform better than the plain model for the large track, where we submitted predictions of equal condition (i.e. single models), supports our assumption.

For runID 2 and 5 models, we utilized both training and development dataset as training material for the challenge main track (please see the Experiments section for details). Since we were using the ensemble method, we assume that the ensembled model is trained on the knowledge of both train and develop datasets. Submission names under ‘2-RoBERTaLarge-10-traindev’ (runID 2) and ‘4-RoBERTaLarge_CTD-5-traindev’ (runID 4) were trained using this strategy and showed superior performance than models only trained on the training dataset. This would suggest that increasing the size of the training set further still would yield additional gains.

‘5-Best-CTD’ (runID 5) was an ensemble of 10 checkpoints across all settings and steps that showed the best performance for the develop dataset.

#### Large-scale text mining subtrack dataset

For the large-track inference, we used single models instead of ensemble methods due to the computational cost. ‘1-BioLM-CTD-lr1e5-filter’ and ‘2-BioLM-CTD-lr5e6-filter’ are models pre-trained with augmented data, and ‘3-BioLM-lr2e5-filter’ is trained without augmented data. The difference between runIDs 1 and 2 is the learning rate during training.

**Figure 6.**
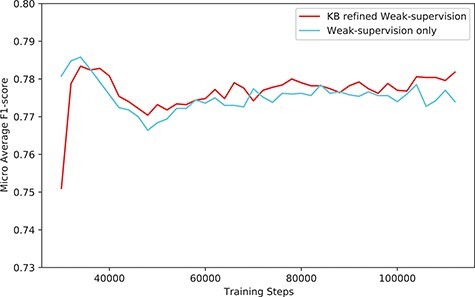
The performance (*F*1-score) of our system (KB-refined weak supervision) and the model trained using simple weak supervision (weak supervision without KB refine).

### Discussion on the proposed weakly supervision method

From Table 4, the overall performances (micro-average) of models trained using our proposed system are better than models without the augmented dataset (denoted as Baseline).

#### Limitation

The overall performance gain using our system was marginal, especially when compared with the model trained with simple weak supervision. Figure 6 shows the performance compared with the model trained with simple weak supervision. The performance using a simple approach was better for the first few steps. However, as training proceeds, the performance of our approach scored higher than weak supervision without KB.

We assume that pretraining the model on the augmented dataset is beneficial for predicting relations with underrepresented training examples. However, our model did not show better performance for the very poorly represented relation types (i.e. <5% of the total dataset). All of our approaches struggled to learn these relationships, suggesting that a minimum number of examples of these types needs to be present in the training data for a machine learning strategy to be viable. Discovering this minimum example number remains a topic of further work.

Similar results are observed for the large-track submission (Table 6). The results for the large track were lower than the expectation based on our experiments on the development dataset. During the challenge, we had a technical issue with our preprocessing system, and this led to the suboptimal performance for predicting at a scale.

**Table 6.** The performance of the model on large-scale text mining subtrack test data (evaluated by the organizers)

	Settings	Performance (test)			
runID (large)	Augmented	*F*1 % (All)	*F*1 % (INHI)	*F*1 % (DIR)	*F*1 % (SUBS)
1	O	75.76	85.22	66.98	65.42
2	O	75.82	84.66	66.91	64.07
3	X	75.18	84.76	65.74	62.98

*Notes:* The base model (language model) for all submissions was BioLM$_\textrm{LARGE}$. The runIDs of the large-track models do not correspond to the runIDs of the main track models.

## Related works

### Biomedical relation extraction

Hand-labelled BioRE datasets are essential and valuable resources for BioRE researches as they provide high-quality annotations. The DDI corpus (26) is a dataset about the drug–drug interaction and developed for the SemEval 2013-DDIExtraction 2013 task (27). The dataset is created from 792 DrugBank texts and 233 MEDLINE abstracts (http://www.mavir.net/resources/179-ddicorpus). The chemical–protein interaction (ChemProt) (19) is a rich resource for chemical–protein interactions, annotated on 2432 PubMed abstracts: train, dev and test datasets of 1020, 612 and 800 abstracts, respectively.

### Distant supervision for relation extraction

Given the cost associated with creating hand-labelled BioRE datasets, researchers have turned to distant supervision approaches for training and evaluating relation extraction models. An example of a distantly labelled dataset is the Genetic Association Database corpus (GAD) (10), generated using the Genetic Association Archive (https://geneticassociationdb.nih.gov/) database. However, it is important to note that the GAD dataset has suboptimal quality and should be considered as a supplementary measure, as highlighted in recent studies evaluating the performance of language models (4). BEEDS by Wang et al. (28) is one of the recent research studies on BioRE using distant supervision. BEEDS utilizes question answering and distant supervision to mine event structures including relation extraction from PubMed articles.

### Complex BioRE

Recent research has highlighted the limitations of representing certain biomedical relations solely as binary relations involving two entities or within a single sentence. This has led to an increased demand for models and resources capable of handling more complex biomedical relations. Addressing this need, BERT-GT (29) was proposed to utilize Graph Transformer with the BERT model to predict cross-sentence n-ary relation (i.e. relations with multiple entities) extraction. The other work by Giorgi et al. (30) proposed a generative approach for relation extraction, enabling the model to naturally predict n-ary relations and cross-sentence relations. Another notable effort in modeling complex biomedical relations is BioRED (16). BioRED focuses on annotating relations among six commonly described entities, namely genes, diseases, chemicals, variants, species and cell lines. The annotated relations in BioRED can be asserted within or across sentence boundaries, necessitating machine reading across entire documents.

### Other models from the BioCreative VII challenge—DrugProt track

In the BioCreative VII challenge—DrugProt track, the Humboldt team’s approach (31) detected drug–protein relations in scientific abstracts using pretrained transformer-based language models and additional side information from biomedical KBs. The NLM-NCBI team (32), on the other hand, employed a sequence labeling framework for drug–protein relation extraction, improving efficiency and performance by recognizing all relevant entities simultaneously.

### Model-generated label-based weak supervision

In the absence of labeled data, a potential method is to employ weak supervision to automatically generate labels from domain KBs. One such example is a work by Shang et al. (33) where they match segments of unlabelled biomedical documents to a biomedical dictionary to create weakly labeled data. Jiang et al. (12) pointed out the shortcomings of model-generated labels and proposed noise-aware continual pretraining for biomedical NER task. Another example of this example is a work by Yoon et al. (13) where they used model-generated weakly labelled dataset to train the final model and showed better generalizability.

## Limitation and further works

Our primary objective in this study was to develop a BioRE system specifically designed for the DrugProt dataset. The DrugProt dataset is characterized by relations that exist within a sentence and involve two entities. The entity types in this dataset are defined as drug and protein.

As a future direction of our research, we intend to extend our approach to encompass relation extraction datasets that involve multiple entity types, such as the BioRED dataset. By incorporating datasets with diverse entity types, we aim to enhance the versatility and applicability of our approach.

## Conclusion

In this paper, we present a BioRE system that utilizes a language model structure and a combination of weak supervision methods, known as KB-refined weak supervision, to enhance its performance. Our system comprises three phases: the preparation of weakly labelled data, initial training of the model on weakly labelled data and fine-tuning using human-labelled data. We developed the weakly labelled dataset by combining two approaches. First, we trained a model on the original dataset to predict labels on external literature, generating a model-labelled dataset. Subsequently, we refined the model-labelled dataset using an external KB.

Our experiments demonstrated that our approach, which employs refined weak supervision, exhibited significant performance gains over models trained using standard human-labelled datasets.

Our source code and automatically annotated drug–protein relation predictions on 31 million entity pairs (based on the DrugProt large task data) are available online for further research, such as for building knowledge graphs.

## Supplementary Material

baad054_Supp

## Data Availability

The materials, including trained weights and automatically annotated relation predictions, are freely available at http://wonjin.info/biore-yoon-et-al-2022.
