# Supplementary material for: Biomedical relation extraction with knowledge base–refined weak supervision
Source: Database (Oxford). 2023 Jul 26;2023:baad054. doi: 10.1093/database/baad054 (PMC10407973; doi:10.1093/database/baad054)
Supplement: baad054_Supp [file baad054_supp.zip › suppl_data/DATABASE_appendix.pdf]

Appendix of
Biomedical relation extraction with knowledge base refined weak-supervision

1 Introduction

This appendix contains information on hyperparameter settings and the training log.

2 Hyperparameter settings

The hyperparameter settings employed in our study are presented below.

For the training of models on BC7-DrugProt datasets (including fine-tuning): learning rate=5e-06, epochs=200 for phase 1 and epochs=100 for phase 3

For the training of models on weakly datasets: learning rate=1e-05, epochs=4

For all experiments: max sequence length=192, batch size=32, warmup ratio=0.05. Seeds were from 0 to 4 or to 9. We used [‘ENToGENEoMK’, ‘KMoGENEoTNE’, ‘ENToCHEMoMK’, ‘KMoCHEMoTNE’] as the markers for the entity and assigned these tokens to the additional special tokens key of the tokenizer.

When language model is ”RoBERTa-large-PM-M3-Voc-hf”: additional details on the model are: vocab size: 50005, hidden size: 1024.

We also made reproducible source code including default hyperparameter settings, pre-processed data and instructions publicly available on our GitHub repository: https://github.com/dmis-lab/BioRE-drugprot-kuaz

3 Training logs

The training log of our system (when the random seed is 0), denoted as *BC7DP-supervised (Transferred)* in the main manuscript, is presented below. The log includes information on the training loss and the learning rate, which is adjusted using the learning rate scheduler.

$\{'loss' : 0.5408, 'learning\_rate' : 4.939979252087142e^{-07}, 'epoch' : 0.99\}$
$\{'loss' : 0.3124, 'learning\_rate' : 9.879958504174283e^{-07}, 'epoch' : 1.98\}$

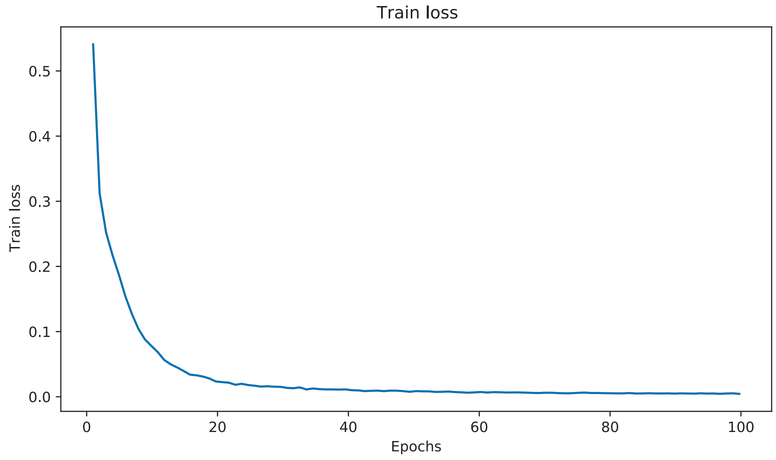

Figure 1: Training loss

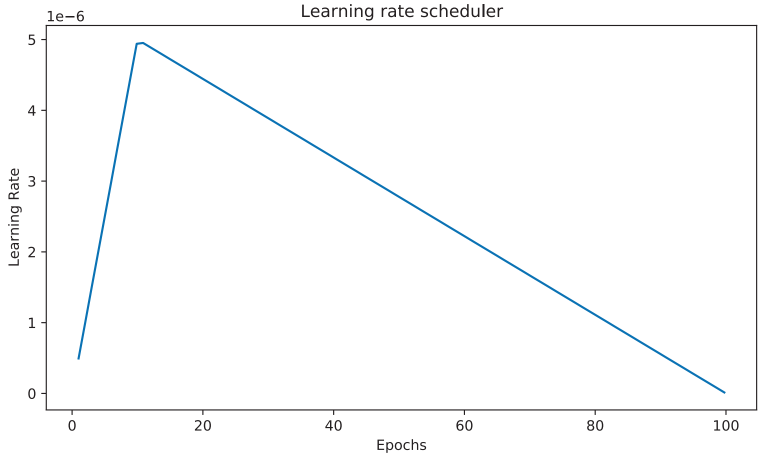

Figure 2: Learning rate

$\{'loss' : 0.2525,' learning\_rate' : 1.4819937756261424e^{-06},' epoch' : 2.96\}$
$\{'loss' : 0.2174,' learning\_rate' : 1.9759917008348566e^{-06},' epoch' : 3.95\}$
$\{'loss' : 0.1868,' learning\_rate' : 2.469989626043571e^{-06},' epoch' : 4.94\}$
$\{'loss' : 0.1538,' learning\_rate' : 2.9639875512522847e^{-06},' epoch' : 5.93\}$
$\{'loss' : 0.1272,' learning\_rate' : 3.4579854764609994e^{-06},' epoch' : 6.91\}$

$\{'loss' : 0.1044,' learning\_rate' : 3.951983401669713e^{-06},' epoch' : 7.9\}$
$\{'loss' : 0.0882,' learning\_rate' : 4.445981326878427e^{-06},' epoch' : 8.89\}$
$\{'loss' : 0.0781,' learning\_rate' : 4.939979252087142e^{-06},' epoch' : 9.88\}$
$\{'loss' : 0.0686,' learning\_rate' : 4.951798833515311e^{-06},' epoch' : 10.86\}$
$\{'loss' : 0.0565,' learning\_rate' : 4.8969312564126486e^{-06},' epoch' : 11.85\}$
$\{'loss' : 0.0498,' learning\_rate' : 4.842063679309986e^{-06},' epoch' : 12.84\}$
$\{'loss' : 0.045,' learning\_rate' : 4.787196102207323e^{-06},' epoch' : 13.83\}$
$\{'loss' : 0.0396,' learning\_rate' : 4.73232852510466e^{-06},' epoch' : 14.81\}$
$\{'loss' : 0.0339,' learning\_rate' : 4.677460948001998e^{-06},' epoch' : 15.8\}$
$\{'loss' : 0.0329,' learning\_rate' : 4.622593370899335e^{-06},' epoch' : 16.79\}$
$\{'loss' : 0.031,' learning\_rate' : 4.567725793796672e^{-06},' epoch' : 17.78\}$
$\{'loss' : 0.028,' learning\_rate' : 4.512858216694009e^{-06},' epoch' : 18.77\}$
$\{'loss' : 0.0234,' learning\_rate' : 4.457990639591347e^{-06},' epoch' : 19.75\}$
$\{'loss' : 0.0225,' learning\_rate' : 4.403123062488684e^{-06},' epoch' : 20.74\}$
$\{'loss' : 0.0216,' learning\_rate' : 4.348255485386021e^{-06},' epoch' : 21.73\}$
$\{'loss' : 0.0184,' learning\_rate' : 4.293387908283359e^{-06},' epoch' : 22.72\}$
$\{'loss' : 0.0199,' learning\_rate' : 4.238520331180695e^{-06},' epoch' : 23.7\}$
$\{'loss' : 0.018,' learning\_rate' : 4.183652754078033e^{-06},' epoch' : 24.69\}$
$\{'loss' : 0.0169,' learning\_rate' : 4.12878517697537e^{-06},' epoch' : 25.68\}$
$\{'loss' : 0.0156,' learning\_rate' : 4.0739175998727074e^{-06},' epoch' : 26.67\}$
$\{'loss' : 0.0161,' learning\_rate' : 4.019050022770045e^{-06},' epoch' : 27.65\}$
$\{'loss' : 0.0154,' learning\_rate' : 3.964182445667382e^{-06},' epoch' : 28.64\}$
$\{'loss' : 0.0152,' learning\_rate' : 3.9093148685647195e^{-06},' epoch' : 29.63\}$
$\{'loss' : 0.0136,' learning\_rate' : 3.854447291462057e^{-06},' epoch' : 30.62\}$
$\{'loss' : 0.0131,' learning\_rate' : 3.799579714359394e^{-06},' epoch' : 31.6\}$
$\{'loss' : 0.0144,' learning\_rate' : 3.7447121372567312e^{-06},' epoch' : 32.59\}$
$\{'loss' : 0.0112,' learning\_rate' : 3.689844560154068e^{-06},' epoch' : 33.58\}$
$\{'loss' : 0.0127,' learning\_rate' : 3.634976983051406e^{-06},' epoch' : 34.57\}$
$\{'loss' : 0.0117,' learning\_rate' : 3.580109405948743e^{-06},' epoch' : 35.56\}$
$\{'loss' : 0.0113,' learning\_rate' : 3.5252418288460803e^{-06},' epoch' : 36.54\}$
$\{'loss' : 0.0113,' learning\_rate' : 3.4703742517434177e^{-06},' epoch' : 37.53\}$
$\{'loss' : 0.011,' learning\_rate' : 3.415506674640755e^{-06},' epoch' : 38.52\}$
$\{'loss' : 0.0113,' learning\_rate' : 3.360639097538092e^{-06},' epoch' : 39.51\}$
$\{'loss' : 0.0101,' learning\_rate' : 3.3057715204354298e^{-06},' epoch' : 40.49\}$
$\{'loss' : 0.0098,' learning\_rate' : 3.2509039433327667e^{-06},' epoch' : 41.48\}$
$\{'loss' : 0.0087,' learning\_rate' : 3.196036366230104e^{-06},' epoch' : 42.47\}$
$\{'loss' : 0.0091,' learning\_rate' : 3.141168789127441e^{-06},' epoch' : 43.46\}$
$\{'loss' : 0.0094,' learning\_rate' : 3.086301212024779e^{-06},' epoch' : 44.44\}$
$\{'loss' : 0.0086,' learning\_rate' : 3.0314336349221158e^{-06},' epoch' : 45.43\}$
$\{'loss' : 0.0094,' learning\_rate' : 2.9765660578194527e^{-06},' epoch' : 46.42\}$
$\{'loss' : 0.0093,' learning\_rate' : 2.92169848071679e^{-06},' epoch' : 47.41\}$
$\{'loss' : 0.0086,' learning\_rate' : 2.8668309036141275e^{-06},' epoch' : 48.4\}$
$\{'loss' : 0.0076,' learning\_rate' : 2.811963326511465e^{-06},' epoch' : 49.38\}$
$\{'loss' : 0.0087,' learning\_rate' : 2.7570957494088018e^{-06},' epoch' : 50.37\}$
$\{'loss' : 0.0083,' learning\_rate' : 2.7022281723061396e^{-06},' epoch' : 51.36\}$
$\{'loss' : 0.0082,' learning\_rate' : 2.6473605952034765e^{-06},' epoch' : 52.35\}$

$\{'loss' : 0.0074,'learning\_rate' : 2.592493018100814e^{-06},'epoch' : 53.33\}$
$\{'loss' : 0.0076,'learning\_rate' : 2.537625440998151e^{-06},'epoch' : 54.32\}$
$\{'loss' : 0.008,'learning\_rate' : 2.4827578638954886e^{-06},'epoch' : 55.31\}$
$\{'loss' : 0.0072,'learning\_rate' : 2.427890286792826e^{-06},'epoch' : 56.3\}$
$\{'loss' : 0.0068,'learning\_rate' : 2.373022709690163e^{-06},'epoch' : 57.28\}$
$\{'loss' : 0.0062,'learning\_rate' : 2.3181551325875003e^{-06},'epoch' : 58.27\}$
$\{'loss' : 0.0067,'learning\_rate' : 2.2632875554848373e^{-06},'epoch' : 59.26\}$
$\{'loss' : 0.0073,'learning\_rate' : 2.2084199783821746e^{-06},'epoch' : 60.25\}$
$\{'loss' : 0.0065,'learning\_rate' : 2.153552401279512e^{-06},'epoch' : 61.23\}$
$\{'loss' : 0.0071,'learning\_rate' : 2.0986848241768494e^{-06},'epoch' : 62.22\}$
$\{'loss' : 0.0069,'learning\_rate' : 2.0438172470741868e^{-06},'epoch' : 63.21\}$
$\{'loss' : 0.0065,'learning\_rate' : 1.9889496699715237e^{-06},'epoch' : 64.2\}$
$\{'loss' : 0.0067,'learning\_rate' : 1.934082092868861e^{-06},'epoch' : 65.19\}$
$\{'loss' : 0.0066,'learning\_rate' : 1.8792145157661984e^{-06},'epoch' : 66.17\}$
$\{'loss' : 0.0063,'learning\_rate' : 1.8243469386635356e^{-06},'epoch' : 67.16\}$
$\{'loss' : 0.0059,'learning\_rate' : 1.769479361560873e^{-06},'epoch' : 68.15\}$
$\{'loss' : 0.0057,'learning\_rate' : 1.7146117844582103e^{-06},'epoch' : 69.14\}$
$\{'loss' : 0.0062,'learning\_rate' : 1.6597442073555475e^{-06},'epoch' : 70.12\}$
$\{'loss' : 0.0061,'learning\_rate' : 1.6048766302528849e^{-06},'epoch' : 71.11\}$
$\{'loss' : 0.0056,'learning\_rate' : 1.550009053150222e^{-06},'epoch' : 72.1\}$
$\{'loss' : 0.0053,'learning\_rate' : 1.4951414760475594e^{-06},'epoch' : 73.09\}$
$\{'loss' : 0.0054,'learning\_rate' : 1.4402738989448966e^{-06},'epoch' : 74.07\}$
$\{'loss' : 0.006,'learning\_rate' : 1.385406321842234e^{-06},'epoch' : 75.06\}$
$\{'loss' : 0.0064,'learning\_rate' : 1.3305387447395713e^{-06},'epoch' : 76.05\}$
$\{'loss' : 0.0058,'learning\_rate' : 1.2756711676369085e^{-06},'epoch' : 77.04\}$
$\{'loss' : 0.0058,'learning\_rate' : 1.2208035905342456e^{-06},'epoch' : 78.02\}$
$\{'loss' : 0.0055,'learning\_rate' : 1.165936013431583e^{-06},'epoch' : 79.01\}$
$\{'loss' : 0.0054,'learning\_rate' : 1.1110684363289202e^{-06},'epoch' : 80.0\}$
$\{'loss' : 0.0052,'learning\_rate' : 1.0562008592262575e^{-06},'epoch' : 80.99\}$
$\{'loss' : 0.0052,'learning\_rate' : 1.0013332821235949e^{-06},'epoch' : 81.98\}$
$\{'loss' : 0.0057,'learning\_rate' : 9.46465705020932e^{-07},'epoch' : 82.96\}$
$\{'loss' : 0.005,'learning\_rate' : 8.915981279182693e^{-07},'epoch' : 83.95\}$
$\{'loss' : 0.0051,'learning\_rate' : 8.367305508156067e^{-07},'epoch' : 84.94\}$
$\{'loss' : 0.0054,'learning\_rate' : 7.81862973712944e^{-07},'epoch' : 85.93\}$
$\{'loss' : 0.0051,'learning\_rate' : 7.269953966102812e^{-07},'epoch' : 86.91\}$
$\{'loss' : 0.0051,'learning\_rate' : 6.721278195076184e^{-07},'epoch' : 87.9\}$
$\{'loss' : 0.0052,'learning\_rate' : 6.172602424049557e^{-07},'epoch' : 88.89\}$
$\{'loss' : 0.0049,'learning\_rate' : 5.62392665302293e^{-07},'epoch' : 89.88\}$
$\{'loss' : 0.0052,'learning\_rate' : 5.075250881996302e^{-07},'epoch' : 90.86\}$
$\{'loss' : 0.005,'learning\_rate' : 4.526575110969675e^{-07},'epoch' : 91.85\}$
$\{'loss' : 0.0048,'learning\_rate' : 3.9778993399430475e^{-07},'epoch' : 92.84\}$
$\{'loss' : 0.0053,'learning\_rate' : 3.4292235689164207e^{-07},'epoch' : 93.83\}$
$\{'loss' : 0.0049,'learning\_rate' : 2.8805477978897933e^{-07},'epoch' : 94.81\}$
$\{'loss' : 0.005,'learning\_rate' : 2.3318720268631657e^{-07},'epoch' : 95.8\}$
$\{'loss' : 0.0045,'learning\_rate' : 1.7831962558365386e^{-07},'epoch' : 96.79\}$
$\{'loss' : 0.005,'learning\_rate' : 1.2345204848099113e^{-07},'epoch' : 97.78\}$

$\{'loss' : 0.0053,' learning\_rate' : 6.858447137832842e^{-08},' epoch' : 98.77\}$
$\{'loss' : 0.0044,' learning\_rate' : 1.3716894275665682e^{-08},' epoch' : 99.75\}$
